# Association of Gly972Arg variant of insulin receptor subtrate-1 and Gly1057Asp variant of insulin receptor subtrate-2 with polycystic ovary syndrome in the Chinese population

**DOI:** 10.1186/s13048-014-0092-4

**Published:** 2014-10-14

**Authors:** Ming-Wei Lin, Mei-Feng Huang, Meng-Hsing Wu

**Affiliations:** Institute of Public Health, National Yang-Ming University, Taipei, Taiwan; Departments of Physiology, National Cheng Kung University College of Medicine, Tainan, Taiwan; Departments of Obstetrics and Gynecology, National Cheng Kung University College of Medicine and Hospital, 138 Sheng-Li Road, 70428 Tainan, Taiwan

**Keywords:** Polycystic ovary syndrome, Polymorphism, *Insulin receptor substrate −1*, *Insulin receptor substrate-2*

## Abstract

**Objective:**

Polycystic ovary syndrome (PCOS) is a common endocrinologic disease in women. In the present study, we examined the relationship of the *IRS-1* Gly972Arg and *IRS-2* Gly1057Asp polymorphisms to PCOS and phenotypic features of PCOS in a Chinese population from Taiwan.

**Materials and methods:**

A total of three hundred and forty genetically unrelated women with age from 18 to 45 years, including two hundred and forty-eight *PCOS* patients and ninety-two control subjects, were recruited. The hormone and biochemical measurements were evaluated for each woman. Genotyping of the *IRS-1* gene Gly972Arg variant and *IRS-2* gene Gly1057Asp variant were performed by using direct sequencing.

**Results:**

We found significant difference in the genotypic distribution of *IRS-2* gene Gly1057Asp between the PCOS group and the control group (p = 0.004). The carriers of homozygous *IRS-2* Asp had an increased risk of PCOS compared with the carriers of Gly/Gly (OR 4.08, 95% C.I. 1.60-10.41, p = 0.003). No significant difference in genotype frequencies of *IRS-1* Gly972Arg was observed between two groups. We further investigated the effect of interaction of *IRS-1* Gly972Arg and *IRS-2* Gly1057Asp on the risk of PCOS and found that women carried *IRS-1* Gly/Arg or *IRS-2* Asp/Asp or carried both *IRS-1* Gly/Arg and *IRS-2* Asp/Asp had a much higher risk of PCOS compared with their counterpart, respectively (OR 2.49, 95% C.I. 1.16-5.37, p = 0.019; OR 11.87, 95% C.I. 1.21-116.84, p = 0.034). We further found, the non-obese PCOS patients carried significantly higher frequency of *IRS-2* Asp/Asp as compared with the control group (p = 0.004). A significant effect of interaction of carrying both *IRS-1* Gly/Arg and *IRS-2* Asp/Asp was also observed in the non-obese PCOS patients (p = 0.003), but not in the obese PCOS patients.

**Conclusions:**

In this study, we found significant association of the variant of *IRS-2* gene as well as the interaction of *IRS-1* and *IRS-2* genes with PCOS, especially in non-obese women. Women with *IRS-2* homozygous Asp variant may be considered as a risk factor for PCOS that needs early detection to prevent further complication in the Chinese population from Taiwan.

## Introduction

Polycystic ovary syndrome (PCOS) is a highly prevalent syndrome of ovarian dysfunction affecting approximately 5-10% of reproductive-aged women [1]. The PCOS is characterized by chronic anovulation, hyperandrogenism, and/or the presence of polycystic ovary morphology. The syndrome demonstrates a significant reproductive and metabolic impact, and is associated with increased risk of type 2 diabetes, dyslipidemia, cardiovascular disease, endometrial carcinoma, and also leads to infertility [2-6]. In general, PCOS can be viewed as a heterogeneous androgen excess disorder with various degrees of gonadotropic and metabolic abnormalities determined by the interaction of multiple genetic and environmental factors. Insulin resistance, particularly in skeletal muscle and adipose tissue with sensitivity in ovarian tissue, affects up to 70% of women with PCOS and is a risk factor in PCOS women for developing type 2 diabetes [7,8]; however, the mechanisms for defects in insulin signaling in the disorder are complex [9] and have not been fully elucidated.

The insulin receptor is a heterotetramer consist of two α, β-dimers. The α-subunit contains the ligand-binding site, while the β-subunit contains a ligand-activated tyrosine kinase. Once tyrosine is phosphorylated, the insulin receptor phosphorylates two intracellular substrates, insulin receptor substrate-1 (IRS-1) and insulin receptor substrate-2 (IRS-2). The IRS-1 serves as a docking molecule for signaling and will activate the enzyme phosphatidylinositol 3-kinase (PI3K), a necessary step for the initiation of several effects of insulin such as glucose transport. When the IRS-1 is dysfunctional, the IRS-2 is the main docking protein for the intracellular propagation of the insulin signal [10]. However, the IRS-2 requires a higher insulin concentration for activation, the hallmark of insulin resistance.

The *IRS-1* gene is located on chromosome 2q36 and encodes a 1,242-amino acid protein with a molecular weight of 131.6 kDa. The most common variant, Gly972Arg (rs1801278), was reported to be associated with insulin resistance, type 2 diabetes and PCOS [11-14]. The *IRS-2* gene is located on chromosome 13q34 and encodes a protein of 1,354 amino acids. Moreover, the most common variant Gly1057Asp (rs1805097) in the *IRS-2* gene has also been reported to influence the susceptibility to insulin resistance and type 2 diabetes in PCOS women [15-17]. Two recent meta-analysis of PCOS studies reported that the *IRS-1* Gly972Arg polymorphism concerning the Gly/Arg *vs*. Gly/Gly genotype is significantly associated with the risk of developing PCOS and that this association is primarily mediated by increasing the levels of fasting insulin [18,19]. However, another meta-analysis of five studies with 519 cases and 883 controls failed to demonstrate significant association between *IRS-2* Gly1057Asp polymorphism and PCOS [19].

Although the meta-analysis reported positive associations of the *IRS-1* Gly972Arg with PCOS and no association between the *IRS-2* Gly1057Asp polymorphism and PCOS, the results from different studies were controversial and were lack of data from Chinese ethnic origin. The aim of the study was to investigate if *IRS-1* Gly972Arg and *IRS-2* Gly1057Asp influence insulin resistance and are associated with risk of PCOS in the Chinese PCOS patients and controls from Taiwan.

## Materials and methods

### Subjects

A total of three hundred and forty genetically unrelated women with age from 18 to 45 years, including two hundred and forty-eight PCOS patients and ninety-two control subjects, were recruited from the Obstetrics and Gynecology clinics of the National Cheng Kung University Hospital (Tainan, Taiwan). This study was approved by the Institution Review Board of the hospital. Written informed consent was obtained from all participants. The study was in compliance with the Helsinki Declaration. All the participants are Han Chinese from the same geographical region in Taiwan.

The diagnosis of PCOS was assigned according to the 2003 Rotterdam criteria (The Rotterdam ESHRE/ASRM-sponsored PCOS consensus workshop group, 2004). The criteria are as follows: (i) oligo- and/or anovulation that is defined as the absence of menstruation for more than 35 days; (ii) clinical and/or biochemical signs of hyperandrogenism: the former is defined as and modified Ferriman-Gallwey score of 6 or greater with/without acne or androgenic alopecia, and the latter as total testosterone level of more than 0.95 ng/ml; and (iii) polycystic ovarian morphology identified by ultrasound scan. Women fulfilled any two of above three criteria were diagnosed as PCOS. The exclusion criteria included non-classic congenital adrenal hyperplasia, hyperprolactinemia, and androgen-secreting tumors. All the patients did not take any medication having effect on insulin levels or hormonal medications, including contraceptive pills, at least two months before their participating in the study. The control patients were enrolled from infertility clinic prior to entering an *in vitro* fertilization program due to tubal and/or male factors with free of menstrual cycle irregularities, clinical or biochemical hyperandrogenism, polycystic ovaries on ultrasound examination, or history of systemic/endocrine disease.

### Hormone and biochemical measurement

The blood samples were obtained in the early follicular phase of menstrual cycle, but the random blood samples were obtained when amenorrheic. Biochemical assessment consisted of complete hormonal, including serum follicle stimulating hormone (FSH) luteinizing hormone (LH), thyroid-stimulating hormone (TSH), prolactin (PRL), estradiol (E_2_), 17-OH-progesterone (17-OHP), total testosterone, and sex-hormone binding globulin (SHBG), and metabolic evaluation, including evaluation of lipid, glucose and insulin levels. All subjects received a 75-g glucose monohydrate in 350-ml water after a 10-h overnight fasting. A total of 5 ml blood sample was drawn before glucose loading and another 5 ml blood samples was drawn at 120 minutes after the glucose loading. Plasma glucose and insulin concentrations were determined by a glucose oxidase method in a glucose analyzer (model 2300, YSI, Yellow Springs, OH, USA) and by an automated chemiluminescence system (ADVIA Centaur Immunoassay System, Siemens Healthcare Diagnostics, Deerfield, IL, USA), respectively. Insulin resistance was evaluated using the homeostasis model analysis (HOMA) [fasting glucose (mg/dL) × fasting insulin (μU/mL)/405], quantitative insulin-sensitivity check index (QUICKI) [1/[log(fasting insulin) + log(fasting glucose)], and the fasting glucose-to-insulin ratio (A/I).

### Genotyping by re-sequencing

A total of 10 mL whole blood sample was taken from each subject for genotyping. Genomic deoxyribonucleic acid (DNA) was extracted from whole blood using the GeneMark extraction Kit (GeneMark Technology Co.,Ltd., Tainan, Taiwan, ROC) according to the manufacturer’s instruction. Genotypings of the *IRS-1* gene Gly972Arg variant (rs 1801278) and the *IRS-2* gene Gly1057Asp (rs1805097) variant were performed by using direct sequencing. The primer sequences for the *IRS-1* Gly972Arg polymorphism were 5’-GGGTCGAGATGGGCAGACT-3’ and 5’-GGGACAACTCATCTGCATGGT-3’; while for the *IRS-2* Gly1057Asp polymorphism were 5’-GGAGCTGTACCGCCTGCC-3’ and 5’-ACCAAAAGCCATCTCGGTGT-3’, respectively. Twenty to fifty nanograms of total genomic DNA was amplified in a total volume of twenty-five microliter containing 900 nM primers, and 12.5 ul of Taq-Man universal PCR master mix (Perkin-Elmer, Applied Biosystems Division) by using the standard polymerase chain reaction (PCR) techniques. The PCR amplification was performed with the following conditions: 95°C, 10 min; followed by 40 cycles of 95°C 1 min, 53°C 30 secs., and 72°C 30 secs. The final step was 72°C for 10 minutes. The PCR reactions were performed in 96-well microtiter plates and the sequencing reactions were performed using the ABI BigDye Terminator reagents (Applied Biosystems, Foster City, CA). The PCR products were sent to the Nucleic Acid Sequencing Center of National Cheng-Kung University for sequencing by using the ABI 3100 DNA sequencer (Applied Biosystems, Foster City, CA). The sequence data were analyzed by using the PolyPhred software (v5.04) [20]. The genotypes were assigned to the subjects independently by two individuals blinded to the subject information.

### Statistical analysis

Data were expressed as mean ± SD or number (%). Two-sample *t* test was applied to compare the mean differences between groups. Differences in genotypic frequencies and categorical data between groups were compared by using Pearson’s chi-squared tests. Hardy-Weinberg equilibrium at each SNP was tested using Pearson’s chi-squared tests. Logistic regression analyses were performed to examine the differences in genotypic frequencies and interaction of two SNPs between the PCOS and control groups. Odds ratios and 95% confidence interval (ORs ± 95% CI) from the logistic regression model after controlling for other covariates were used to estimate the magnitude of the association between genotype and PCOS. The statistical analyses were performed using the SPSS program (Version 17.0, SPSS Inc., Chicago, IL, USA). A *p* value less than 0.05 was considered as statistically significant.

## Results

### Clinical and biochemical characteristics of the study population

The clinical and biochemical characteristics of the PCOS and control groups were summarized in Table 1. The PCOS subjects were significant younger and had higher body weight and body mass index as compared with the control subjects. As we could expect, the levels of LH, testosterone, free androgen index, SHBG and Hirsutism score were significantly higher in the PCOS group than in the control group. Moreover, the PCOS patients also had significantly elevated levels of glucose, insulin at 2 hour during the oral glucose tolerance test, and HbA1C (glucose: 110.4 ± 36.2 mg/dL; insulin: 54.8 ± 52.0 mg/dL; HbA1C: 5.5 ± 0.5 (%) ) compared with the control subjects (glucose: 99.0 ± 25.9 mg/dL; insulin: 32.5 ± 26.5 mg/dL; HbA1C: 5.3 ± 0.3 (%)). The results remained significant after adjusting the age effect between the PCOS and control groups.

**Table 1:** **Descriptive characteristics of the study participants**

	**PCOS group**	**Control group**	***P*** ^***a***^
Subjects (n)	248	92	
Age (years)	28.2 ± 5.4	32.4 ± 5.5	< 0.001
Body weight (kg)	62.7 ± 14.8	56.9 ± 10.3	< 0.001
Body height (cm)	159.4 ± 5.3	159.7 ± 5.3	0.681
BMI (kg/m^2^)	24.7 ± 5.5	22.3 ± 4.0	< 0.001
Waist circumference (cm)	83.9 ± 13.5	82.7 ± 12.2	0.705
Systolic BP (mmHg)	117 ± 15	115 ± 14	0.178
Diastolic BP (mmHg)	70 ± 11	70 ± 11	0.852
Total cholesterol (mg/dL)	181.9 ± 43.0	189.9 ± 35.5	0.285
Triglycerides (mg/dL)	104.3 ± 67.5	91.6 ± 46.0	0.258
HDL-C (mg/dL)	53.8 ± 12.8	58.6 ± 17.4	0.063
LDL-C (mg/dL)	114.4 ± 43.8	115.3 ± 32.2	0.914
Fasting insulin	9.9 ± 10.5	9.3 ± 15.5	0.733
2 hr insulin	54.8 ± 52.0	32.5 ± 26.5	< 0.001
HbA1C (%)	5.5 ± 0.5	5.3 ± 0.3	0.037
AC (mg/dL)	89.4 ± 12.0	87.6 ± 5.3	0.086
PC (mg/dL)	110.4 ± 36.2	99.0 ± 25.9	0.036
HOMA index (mg/L)	2.3 ± 2.6	2.0 ± 3.4	0.542
A/I (AC/ Fasting Insulin)	16.7 ± 11.7	17.9 ± 11.4	0.489
QUICKI index (mg/L)	3.4 ± 0.8	3.5 ± 0.7	0.674
SHBG (nmol/L)	37.8 ± 29.7	59.6 ± 53.8	0.007
17-OHP (ng/mL)	1.8 ± 1.4	2.0 ± 1.3	0.529
Hirsutism score	5.6 ± 3.8	3.2 ± 2.9	0.016
TSH (μU/mL)	2.1 ± 1.3	2 .0 ± 1.0	0.618
LH (mIU/mL)	8.0 ± 5.3	4.3 ± 2.5	< 0.001
FSH (mIU/mL)	5.6 ± 2.0	6.0 ± 2.4	0.212
E2 (pg/mL)	45.8 ± 22.9	43.1 ± 27.5	0.484
Testosterone (ng/mL)	0.55 ± 0.31	0.35 ± 0.19	< 0.001
Free androgen index	5.98 ± 4.61	4.04 ± 4.02	0.043
PRL (ng/mL)	12.5 ± 6.4	13.5 ± 6.3	0.248

^a^Compared by t test.

### *IRS-1* and *IRS-2* genotypes and gene-gene interaction

There was significant difference in *IRS-2* gene Gly1057Asp genotypic distribution between the PCOS group and the control group (chi-squared test p = 0.004). The carriers of homozygous *IRS-2* Asp had an increased risk of PCOS compared with the carriers of Gly/Gly after adjusting for age and BMI (OR =4.08, 95% C.I. 1.60-10.41, p = 0.003) (Table 2). However, no significant difference in genotype frequencies of *IRS-1* Gly972Arg was observed between two groups (Table 2). The *IRS-1* Gly972Arg variant was in Hardy-Weinberg equilibrium, but the *IRS-2* Gly1057Asp variant was not in Hardy-Weinberg equilibrium in both PCOS and control groups.

**Table 2:** **Genotypic distribution of polymorphisms in the** ***IRS1*** **and** ***IRS2*** **genes between the PCOS group and control group**

	**PCOS group**	**Control group**	***p*** ^***a***^	**OR (95% C.I.)**	***p*** ^***b***^
*IRS-1 Gly972Arg*	(n = 248)	(n = 92)			
GG	220 (88.7%)	84 (91.3%)	0.622	1	
GA	28 (11.3%)	8 (8.7%)		1.03 (0.42-2.53)	0.942
*IRS-2 Gly1057Asp*	(n = 95)	(n = 74)			
GG	12 (12.6%)	25 (33.8%)		1	
GA	18 (18.9%)	13 (17.6%)	0.004	2.77 (0.87-8.81)	0.084
AA	65 (68.4%)	36 (48.6%)		4.08 (1.60-10.41)	0.003
GG + GA	30 (31.6%)	38 (51.4%)	0.015	1	
AA	65 (68.4%)	36 (48.6%)		2.52 (1.21-5.26)	0.014
*IRS-1* and *IRS-2*	(n = 95)	(n = 74)			
GG/GG + GA	26 (27.4%)	35 (47.3%)		1	
GA/AA	58 (61.1%)	38 (51.4%)	0.003	2.49 (1.16-5.37)	0.019
GA and AA	11 (11.6%)	1 (1.4%)		11.87 (1.21-116.84)	0.034

^a^Compared by chi-square test.

^b^Compared by logistic regression after adjusting for age and BMI.

When we further investigated the effect of interaction of *IRS-1* Gly972Arg and *IRS-2* Gly1057Asp on the risk of PCOS, we found that women carried *IRS-1* Gly/Arg or *IRS-2* Asp/Asp had an increased risk of PCOS (OR = 2.49, 95% C.I. 1.16-5.37, p = 0.019). Moreover, carriers of both *IRS-1* Gly/Arg and *IRS-2* Asp/Asp had a much higher risk of PCOS compared with their counterpart (OR = 11.87, 95% C.I. 1.21-116.84, p = 0.034) (Table 2).

### *IRS-2* genotype and clinical phenotypes

We then evaluated the association between *IRS-2* Gly1057Asp genotype and clinical phenotypes in all subjects of both PCOS (n = 95) and control (n = 74) groups and found the levels of fasting insulin, and HOMA index were significantly higher in women carrying the homozygous Asp/Asp genotype than their counterparts (Gly/Gly and Gly/Asp) (Table 3). However, when we focused on the PCOS group, the association between *IRS-2* genotype and clinical phenotypes became statistically insignificant (data not shown).

**Table 3:** **Relationship between clinical phenotypes and genotypes of the** ***IRS-2 Gly1057Asp*** **polymorphism**

	**GG + GA**	**AA**	***p*** ^***a***^
Subjects (n)	68	101	
Age (years)	30.0 ± 6.2	29.7 ± 6.0	0.688
Body weight (kg)	58.7 ± 13.4	61.3 ± 13.3	0.208
Body height (cm)	159.8 ± 5.3	160.1 ± 5.6	0.779
BMI (kg/m^2^)	23.0 ± 5.0	23.9 ± 4.8	0.219
Waist circumference (cm)	80.8 ± 12.1	86.2 ± 11.6	0.058
Systolic BP (mmHg)	113 ± 12	117 ± 15	0.055
Diastolic BP (mmHg)	69 ± 10	71 ± 11	0.209
Total cholesterol (mg/dL)	181.7 ± 48.3	180.6 ± 45.5	0.908
Triglycerides (mg/dL)	91.3 ± 59.5	110.8 ± 68.3	0.152
HDL-C (mg/dL)	55.6 ± 12.0	54.3 ± 13.6	0.653
LDL-C (mg/dL)	111.2 ± 33.4	114.7 ± 28.3	0.598
Fasting insulin	6.7 ± 6.1	11.0 ± 15.3	0.025
2 hr insulin	38.3 ± 31.6	54.2 ± 56.7	0.087
HbA1C (%)	5.4 ± 0.3	5.5 ± 0.5	0.544
AC (mg/dL)	87.6 ± 5.8	90.4 ± 16.8	0.164
PC (mg/dL)	105.4 ± 29.9	109.3 ± 40.2	0.557
HOMA index (mg/L)	1.5 ± 1.6	2.6 ± 3.8	0.022
A/I (AC/ Fasting Insulin)	19.1 ± 11.3	17.4 ± 12.3	0.427
QUICKI index (mg/L)	3.6 ± 0.8	3.5 ± 0.8	0.454
SHBG (nmol/L)	48.5 ± 41.9	40.2 ± 42.5	0.337
17-OHP (ng/mL)	1.8 ± 1.3	2.0 ± 1.4	0.400
Hirsutism score	4.6 ± 3.3	5.8 ± 3.9	0.289
TSH (μU/mL)	2.4 ± 1.5	2.0 ± 0.9	0.044
LH (mIU/mL)	6.8 ± 4.5	7.6 ± 5.5	0.367
FSH (mIU/mL)	5.6 ± 2.3	6.0 ± 2.3	0.445
E2 (pg/mL)	42.5 ± 23.4	48.2 ± 25.5	0.217
Testosterone (ng/mL)	0.4 ± 0.3	0.5 ± 0.3	0.289
Free androgen index	4.60 ± 3.61	6.01 ± 4.61	0.217
PRL (ng/mL)	12.8 ± 6.5	12.3 ± 6.1	0.692

^a^Compared by t test.

### *IRS-1* and *IRS-2* genotypes and obesity

When the study subjects were classified into obese (obese: BMI ≥ 27 kg/m^2^) (PCOS n = 72; Control n = 9) and non-obese groups (BMI < 27 kg/m^2^) (PCOS n = 176; Control n = 83), the non-obese PCOS patients carried significantly higher frequency of *IRS-2* Asp/Asp as compared with the control group (p = 0.004) (Table 4). A significant effect of interaction of carrying both *IRS-1* Gly/Arg and *IRS-2* Asp/Asp was also observed in the non-obese PCOS patients (p = 0.003), but not in the obese PCOS patients (p = 0.834).

**Table 4:** **Genotypic distribution of polymorphisms in the** ***IRS1*** **and** ***IRS2*** **genes between the control group and the PCO group stratified by BMI**

	**Non-obese group (BMI <27)**			**Obese group (BMI ≥ 27)**		
	**PCOS group**	**Control group**	***p*** ^***a***^	**PCOS group**	**Control group**	***P*** ^***b***^
	**(n = 176)**	**(n = 83)**		**(n = 72)**	**(n = 9)**	
*IRS-1 Gly972Arg*						
GG	156 (88.6%)	77 (92.8%)	0.792	64 (88.9%)	7 (77.8%)	0.307
GA	20 (11.4%)	6 (7.2%)		8 (11.1%)	2 (22.2%)	
*IRS-2 Gly1057Asp*	(n = 70)	(n = 68)		(n = 25)	(n = 6)	
GG	8 (11.4%)	24 (35.3%)	0.004	4 (16.0%)	1 (16.7%)	1.000
GA	15 (21.4%)	12 (17.6%)		3 (12.0%)	1 (16.7%)	
AA	47 (67.1%)	32 (47.1%)		18 (72.0%)	4 (66.7%)	
GG + GA	23 (32.9%)	36 (52.9%)	0.027	7 (28.0%)	2 (33.3%)	1,000
AA	47 (67.1%)	32 (47.1%)		18 (72.0%)	4 (66.7%)	
*IRS-1* and *IRS-2*	(n = 70)	(n = 68)		(n = 25)	(n = 6)	
GG / GG + GA	20 (28.6%)	33 (48.5%)	0.003	6 (24.0%)	2 (3.3%)	0.834
GA / AA	43 (61.4%)	35 (51.5%)		15 (60.0%)	3 (50.0%)	
GA and AA	7 (10.0%)	0 (0.0%)		4 (16.0%)	1 (16.7%)	

^a,b^Comparison between the PCOS group and the control group by chi-square test.

## Discussion

PCOS is a highly prevalent syndrome of ovarian dysfunction and affects up to 10% of reproductive age women, nearly half of whom will develop impaired glucose tolerance or type 2 diabetes [8]. This predisposition to type 2 diabetes is a consequence of defects in both insulin action [9] and insulin secretion [15]. Recently, several polymorphisms in *IRS-1* and *IRS-2* have been implicated in PCOS [16,21]. However, the results in PCOS patients were in considerable disagreement and, therefore, the role of these variants in the pathogenesis of insulin resistance and PCOS remains debatable. In the present study, we found significant association of the variants of *IRS-2* gene as well as the interaction of *IRS-1* and *IRS-2* genes with PCOS, especially in non-obese women. We also found significantly increased levels of fasting insulin, and elevated HOMA index in women carrying the homozygous Asp/Asp genotype than their counterpart (Gly/Asp and Gly/Gly).

In our study, we found women with homozygous *IRS-2* Asp genotypes had a significantly increased risk of PCOS compared with the carriers of homozygous *IRS-2* Gly (OR = 4.08, 95% CI: 1.60-10.41). We also observed women with the *IRS-2* Asp/Asp genotype had significantly higher fasting insulin and HOMA index compared with those with Gly/Asp and Gly/Gly genotypes. However, Ehrmann et al. reported that the *IRS-2* Gly/Gly genotype carriers of their nondiabetic subjects had significantly higher 2-h oral glucose tolerance test glucose levels compared with those with Gly/Asp and Asp/Asp genotypes in whites or Gly/Asp genotype in African-Americans [15]. The susceptibility allele of *IRS-2* Gly1057Asp reported in our population is completely opposite from those reported in other populations. When we further examined the minor allele frequency of *IRS-2* Gly1057Asp in different populations, we observed that the Gly is the minor allele in our population, while it is Asp in Caucasians and African Americans [19]. The increased homozygous *IRS-2* Asp polymorphism in the population from Taiwan might be due to different evolutionary force, such as genetic drift, or selection pressure. It may be also due to different techniques used for genotyping of allelic variants. Our study used PCR following by re-sequencing to conduct our genotyping which will produce more reliable genotypes.

We did not find significant difference in genotype frequencies of *IRS-1* Gly972Arg between the PCOS and control groups. Our finding is contradictory to the results from meta-analysis [19]. This inconclusive results in our population deserved further investigations with larger independent sample in the Chinese population from Taiwan.

We found the *IRS-1* Gly972Arg variant was in Hardy-Weinberg equilibrium, but the *IRS-2* Gly1057Asp variant was not in Hardy-Weinberg equilibrium in both PCOS and control groups. The possible reasons why the *IRS-1* SNP in the control group is in equilibrium while it is not for the *IRS-2* marker may be due to different selection pressure for *IRS-1* and *IRS-2* variants or the *IRS-2* Gly1057Arg variant is a recent mutation.

Our study found significant association of the variants of *IRS-2* gene as well as the interaction of *IRS-1* and *IRS-2* genes with PCOS. The magnitudes of associations were more profound in non-obese women group (Table 4). A couple of studies reported that the association of the Asp1057 allele in *IRS-2* with type 2 diabetes may be mediated by interaction of the polymorphism with obesity on several diabetes-related traits [20,22]. The exact molecular mechanism of *IRS-2* Gly1057Asp polymorphism on insulin action is not clear, but it is speculated that this variant introduces an exchange of a charged amino acid (Asp) with a neutral one (Gly) in the domain of IRS-2 molecule located in between two putative tyrosine phosphorylation sites (at positions 1042 & 1072) of the protein. This could produce alterations in downstream signaling through IRS-2 [22].

In this study, despite the PCOS subjects were significant younger, with higher risk of morphologic change, glucose intolerance, and endocrine dysfunction than the control subjects (Table 1), we only found increased levels of fasting insulin, and elevated HOMA index in women carrying the homozygous Asp/Asp genotype than their counterpart (Gly/Gly and Gly/Asp) (Table 3). It may be probable that women with *IRS-2* homozygous Asp variant are more likely to show early signs of metabolic risks rather than to be associated with the development of PCOS.

In our previous study, the level of interleukin-6, which is considered as an early low-grade chronic inflammatory marker, was increased in PCOS women. But the elevated interleukin-6 level was reduced significantly after metformin treatment, especially among PCOS women with *IRS-2* homozygous Asp variant [23]. In this study, we did not have the information of metformin treatment, thus we are unable to evaluate the effect of metformin treatment.

There are several limitations in the study. Firstly, our control subjects were recruited from outpatient department of the hospital, so they might not be well-presented as in the general population. We were also aware that the inclusion and exclusion criteria of control subjects we applied in the study might result in over-estimation of the odds ratio of the *IRS-2* gene on PCOS [24]. The control subjects of the study were enrolled from infertility clinic prior to entering an in vitro fertilization program due to tubal and/or male factors with free of menstrual cycle irregularities, clinical or biochemical hyperandrogenism, polycystic ovaries on ultrasound examination, or history of systemic/endocrine disease. General speaking, they were normal in endocrine function, but were recruited due to infertility problem. Therefore, they could be viewed as normal in male factor but mechanical reason in tubal factor. The issue of over-estimation of odds ratio might not be as severe as we suspect. Secondly, despite we found that the levels of 2-hour insulin, glucose, and HbA1C in the PCOS group significantly higher than the control group, however, we could not find significant differences in HOMA index, A/I, and QUICK index between two groups. The possible reason is that our PCOS group is significantly younger than the control group. For younger PCOS women, the insulin sensitivity/resistance may be normal or only mild hyperinsulinemia. Thirdly, in the oral glucose tolerance test, we only measured the glucose and insulin levels at the fasting and 2-hour time points, thus may limit us to capture the entire picture of insulin sensitivity/resistance during the time course. Lastly, although significant association of the variant of *IRS-2* gene and its interaction effect with *IRS-1* gene related to PCOS was found, the sample size of our study is relatively small. In order to confirm our findings, further larger well-designed studies, especially in different ethnic populations are warranted.

In summary, we found significant association of the variants of *IRS-2* gene as well as the interaction of *IRS-1* and *IRS-2* genes with PCOS, especially in non-obese women. Women with *IRS-2* homozygous Asp variant may be considered as a risk factor for PCOS that needs early detection to prevent further complications in the Chinese population of Taiwan.
